# Survival Outcomes after Placement of Inferior Vena Cava Filters in Cancer Patients: Insights from a Comprehensive Cancer Center’s Experience

**DOI:** 10.3390/jcm12237209

**Published:** 2023-11-21

**Authors:** Hikmat Abdel-Razeq, Faris Tamimi, Mohammed J. Al-Jaghbeer, Baha’ Sharaf, Rashid Abdel-Razeq, Jafar Bani Issa, Hala Abu-Jaish, Osama Salama

**Affiliations:** 1 Department of Internal Medicine, King Hussein Cancer Center, Amman 11941, Jordan; faris.tamimi@uhn.ca (F.T.); ma.16764@khcc.jo (M.J.A.-J.); bs.13628@khcc.jo (B.S.); abdelr3@ccf.org (R.A.-R.); osamaj.salama@gmail.com (O.S.); 2 School of Medicine, The University of Jordan, Amman 11942, Jordan; 3 Department of Radiology, King Hussein Cancer Center, Amman 11941, Jordan; jb.11084@khcc.jo; 4 School of Medicine, Jordan University of Science and Technology, Irbid 22110, Jordan; hiabujaish17@med.just.edu.jo

**Keywords:** cancer, inferior vena cava, IVC filter, thrombosis, venous thromboembolism, VTE

## Abstract

Background: Inferior vena cava (IVC) filters serve as a vital intervention when systemic anticoagulation proves ineffective or contraindicated, particularly in the context of cancer patients. This study aimed to provide real-world insights into the outcomes of cancer patients following IVC filter placement. Patients and methods: Cancer patients with IVC filters were retrospectively reviewed. The indications and survival outcomes following IVC filter insertion have been reported. Results: A total of 176 cancer patients with IVC filters were included in the study. The median patient age was 56 years (range: 18–88 years). Solid tumors were the most common primary cancers (n = 125, 71.0%), and the majority (n = 99, 79.2%) had the advanced-stage disease at the time of IVC insertion. The filters were inserted because of contraindications to anticoagulation (n = 99, 56.3%) or the failure of anticoagulation (n = 56, 31.8%). The median survival (range) following filter placement was only 2 (1.45–2.55) months for patients with advanced-stage solid tumors, 5 (0.62–9.38) months for patients with brain tumors, and 44 (8.59–79.41) months for those with early-stage solid tumors, *p* < 0.001. Conclusions: Our findings suggest that IVC filter placement offers limited benefits to patients with advanced-stage disease. The underlying tumor, stage, and life expectancy are crucial factors in the decision-making process before IVC filter insertion.

## 1. Introduction

Venous thromboembolism (VTE), which includes both deep vein thrombosis (DVT) and pulmonary embolism (PE), is frequently encountered in cancer patients. The odds of developing VTE in cancer patients are more than six times higher than those in the general population [1]. Additionally, active cancer accounts for nearly 20% of all VTE cases in the community [2]. The risk is considerably higher in certain types of cancers, such as malignant brain tumors and mucinous-secreting adenocarcinomas like ovarian, pancreatic, and gastric cancers [3]. Cancer patients receiving anticancer therapy, including chemotherapy, targeted therapy, immunotherapy, and endocrine therapy, are at a higher risk, especially during the first few months after cancer diagnosis [4,5]. Comorbidities, especially in older cancer patients, also add to this risk. Pressure exerted by the tumor on vascular structures and endothelial injury is frequently encountered in cancer patients. Cancer surgery and poor ambulation during the postoperative period can be serious risk factors for VTE when systemic anticoagulation is hazardous or contraindicated [6].

Venous thromboembolism can be complicated by sudden death due to pulmonary embolism and is considered a leading cause of death in patients [7,8]. Additionally, VTE can lead to long-term complications that may impair the quality of life (QOL) of cancer patients. Such complications may include pulmonary hypertension and chronic venous insufficiency (post-thrombotic syndrome) [9]. Additionally, treatment of VTE with anticoagulants may preclude diagnostic or therapeutic procedures that patients with cancer may need [10]. Thus, VTE may lead to significant delays in anticancer therapy and further increase the escalating costs of cancer therapy.

VTE is usually treated with anticoagulants; however, certain clinical scenarios, such as active bleeding or a high risk of bleeding, may prohibit anticoagulant use [11,12]. Even with adequate anticoagulation, the risk of recurrent VTE is significantly higher in cancer patients compared to those without [13]. This led to the development of the inferior vena cava (IVC) filter, an implantable device designed to intercept a thrombus that has broken free from the lower extremities or pelvis veins to prevent pulmonary embolism (PE). First conceived by Trousseau in 1868 [14], the IVC filter was developed in the 1960s to quickly replace the old surgical techniques for mechanical PE prevention, such as IVC surgical ligation or interruption [15,16]. Currently, IVC filter use has markedly increased to reach levels that many clinicians deem controversial [17,18,19], especially considering that the absolute indications for IVC filters have not expanded beyond adults with confirmed acute PE or proximal DVT with contraindications to anticoagulation, active bleeding, or a high risk of bleeding (Class I, level B evidence) [20]. Moreover, while IVC filters decrease the recurrent PE rate, reviews and meta-analyses do not show any mortality benefits [17,21]. Additionally, IVC filter placement is not without complications, including bleeding, infection, contrast-related toxicities, malpositioning, guidewire entrapments, acute venous thrombosis, hematoma, or arteriovenous fistula formation [22,23,24]. Furthermore, there are long-term complications, such as filter erosion/migration or embolization and chronic thrombosis/recurrent thromboembolism [22,23,24]. Recent technological developments, such as retrievable filters for temporary insertion, are gaining attention because they may mitigate some complications [25,26,27].

All IVC filter-related concerns and the surprising lack of strong evidence in the literature make the use of IVC filters challenging and their value debatable, especially in cancer patients [28]. In this paper, we present the outcomes of IVC filter insertion in cancer patients, particularly in those with advanced-stage disease.

## 2. Methods

This retrospective study was conducted at the King Hussein Cancer Center (KHCC) in Amman, Jordan. This study was approved by the Institutional Review Board (IRB) of KHCC (approval number: 20-KHCC-136). Patients aged 18 years or older with a pathologically confirmed malignancy and IVC filter placed at our center were deemed eligible for this study.

Electronic medical records and radiology databases were reviewed from 2006 to 2021 for all patients discharged with an IVC filter. Clinical data for the patients were collected using case report forms. The following variables were collected for each patient: age at the time of IVC filter insertion; age at cancer diagnosis; site and stage of malignancy; type of anticancer therapy used during the six weeks before IVC filter insertion, including surgical intervention, chemotherapy, and radiation therapy; site of VTE; indication for IVC filter insertion; date of IVC filter insertion; date of death; and date of the last follow-up. The patients were stratified into four groups based on the primary tumor stage: early-stage solid tumors, advanced-stage solid tumors, hematological malignancies, and brain tumors. Advanced-stage cancer was defined as a metastatic spread (stage IV), other than bone-only disease. The procedure was performed by one of our interventional radiologists using the jugular or femoral approach in a microaccess system. Before filter insertion, an IVC venogram was performed to evaluate the anatomy and diameter of the IVC and to exclude the existence or extension of the thrombus in the IVC. Various types of filters were used, including the Option^TM^ ELITE retrievable filter (Argon Medical Devices, Plano, TX, USA), Celect^TM^ Platinum filter (Cook Medical, Bloomington, IN, USA), and ALN filter (Macromed, Lancashire, UK). Retrievable IVC filters are a newer generation of filters that can be removed (retrieved) when the risk of a blood clot embolizing the lungs subsides significantly or when anticoagulants are safe to use. Permanent filters, on the other hand, are placed to stay and cannot be retrieved. Permanent filters are usually used for patients with short anticipated survival or those with long-lasting contraindications for anticoagulation. IVC retrieval helps reduce lower extremity swelling and potential recurrent DVT. However, patients with retrievable filters have a higher risk of developing complications than those with permanent filters. Filter migration, vena cava perforation, and filter fracture are significantly more common with retrievable filters than with permanent ones [29].

Medical records were also reviewed for immediate and delayed complications related to IVC filter insertion, including bleeding, hematoma, filter migration, lower-limb edema, and DVT.

Survival analysis was performed using the Kaplan–Meier method in SPSS Inc. (version 16.0; Harvard University, Cambridge, MA, USA). The primary outcome of this study was patient survival after the IVC filter insertion. Survival time was defined as the time between IVC filter insertion and the occurrence of death and was calculated in months. Descriptive statistics were interpreted as medians, interquartile ranges (IQRs), and percentages. The Shapiro–Wilk test was used to check if the continuous variable followed a normal distribution of continuous data. Survival comparisons were carried out using the log-rank test and were compared based on the type and stage of the tumor.

## 3. Results

During the study period, 176 cancer patients treated and followed up at our institution had IVC filters inserted. The median age (IQR) was 56 (47–65) years, and 86 (48.9%) patients were males. The most common primary cancers were solid tumors (n = 125, 71.0%), brain tumors (n = 28, 15.9%), and hematological malignancies (n = 23, 13.1%). Breast, colorectal, lung, bladder, and pancreatic cancers were the most common solid tumors encountered. At the time of IVC insertion, most patients with solid tumors (n = 99, 79.2%) had advanced-stage disease. Both hematological malignancies and brain tumors were classified as “unstageable” (Table 1).

During the 6 weeks before IVC filter placement, 70 (39.8%) patients were on active chemotherapy, 14 (7.9%) had major cancer-related surgery, and 41 (23.3%) were on hospice and palliative care services. Before IVC filter placement, 122 (69.3%) patients had DVT, with or without PE, whereas 41 (23.3%) had isolated PE. Contraindication to anticoagulation was the main indication for filter placement (n = 99, 56.3%) and was mostly related to active bleeding or a high risk of bleeding, while 56 (31.8%) patients had their filters inserted due to anticoagulation failure, which manifested as recurrent thromboembolic episodes despite adequate anticoagulation. Following IVC filter placement, 83 patients (47.2%) were anticoagulated using unfractionated heparin (UFH) or low-molecular-weight heparin (LMWH). IVC filter insertion was complicated by filter thrombosis (n = 3, 1.7%) and recurrent thrombosis in 19 (10.8%) patients.

**Figure 1:**
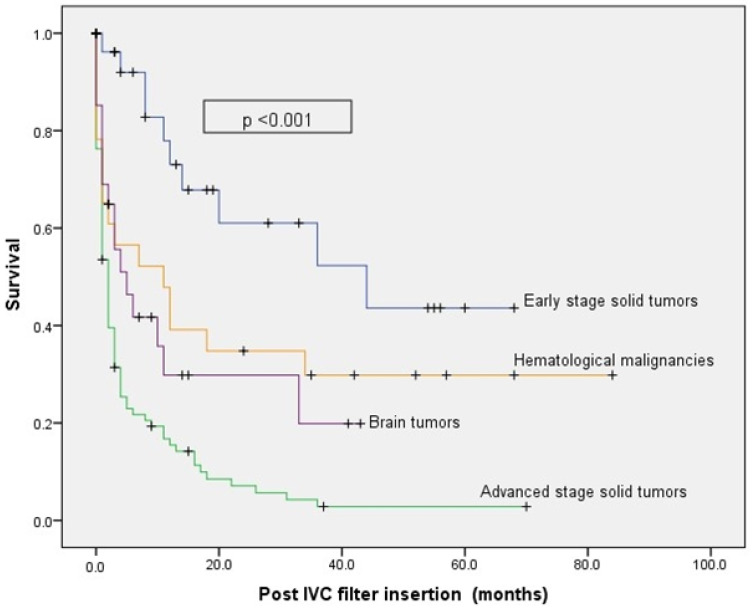
Survival curves post-IVC filter insertion (months). Overall survival of patients following IVC filter insertion according to tumor type and stage. Overall survival was the lowest in patients with advanced-stage disease.

The median survival (range) following filter placement was only two months (1.45–2.55) for patients with advanced-stage solid tumors, five months (0.62–9.38) for patients with brain tumors, and 44 months (8.59–79.41) for those with early-stage solid tumors (*p* < 0.001) (Figure 1). Filters were retrieved from only eight (4.5%) patients. At the end of the follow-up period, 131 deaths were reported, and 45 patients were still alive.

## 4. Discussion

In our study, although most IVC filters were appropriately indicated, the survival following filter placement was poor. In patients with advanced-stage solid tumors, the median survival time is only 2 months. Additionally, the median survival was between 5 and 44 months for patients with brain and early stage tumors, respectively. These numbers are similar to those of a previously published study by our group. This study showed a very limited value of the IVC filters in patients with advanced-stage disease. Among 59 patients with stage IV disease for whom survival data were available, the median survival was only 1.31 (0.92–2.20) months. Notably, 23 patients (39.0%) survived for less than a month, and 40 (67.8%) survived for less than 3 months [30]. Similar conclusions were reached in a study from the University of Pennsylvania, which showed poor survival rates of 68.8% at 30 days, 49.4% at 3 months, and 26.8% at 1 year in patients with all cancer stages [31]. Stratifying patients by cancer stage revealed that the 1-year survival rate for stage I-III cancer was 77.9%, compared to only 13.7% for stage IV disease. It is worth noting that 42 of the 91 stage IV cancer patients died within 6 weeks of the IVC filter placement [31]. Another study from the University of Texas also demonstrated poor outcomes with median survival periods for patients with solid and liquid tumors of 145 days (4.8 months) and 207 days (6.9 months), respectively [32]. Furthermore, the probability of survival at 30 days was 0.76 in patients classified with widely metastatic or disseminated disease [32].

The two largest randomized controlled trials, PREPIC1 [33] and PREPIC2 [34], on IVC filter outcomes had questioned their added benefit on overall survival. Among the 799 patients, only 118 (14.8%) had active cancer, and the stage of the cancer was not specified. Both these studies and others, including a California-based database study of 14,000 cancer patients with VTE, did not show a survival benefit from IVC filter use [35]. An 8-year follow-up study of PREPIC1 showed an increased incidence of DVT in patients with IVC filters [36]. Furthermore, even for recurrent PEs, the PREPIC2 trial did not show a difference between patients treated with a retrievable IVC filter plus anticoagulation and those treated with anticoagulation alone at 3 months [37]. A smaller randomized trial conducted specifically on 64 cancer patients showed no advantage of using the IVC filters in terms of recurrent thrombosis, recurrent pulmonary embolism, or survival [37].

IVC filter use has witnessed a substantive rise since the 1960s, especially in the US, peaking in 2010 [38,39,40,41]. Following the 2010 US Food and Drug Administration (FDA) safety communication, IVC filter utilization dropped significantly [42]. Moreover, IVC filters are inappropriately inserted in several cases. In this study, a nationwide inpatient sample was reviewed in recent years, before and after the FDA safety communication. The authors concluded that the IVC filter placements steadily increased between 2005 (n = 100,434) and 2010 (n = 129,614), with a growth rate of 5.8%. However, IVC filter placement subsequently declined between 2010 and 2014 (n = 96,005), with a decline rate of −6.5% [42].

In a population-based study, the indications for IVC filter placement, as judged by the reviewers, were debatable in 23% and clearly not indicated in 26% of cases, leaving only half with undebatable indications [43]. The low rate of IVC filter retrieval in our study, in only eight (4.5%) patients, may also indicate that the majority of such patients had an anticipated poor outcome; thus, retrieving the IVC filter was not attempted. 

A recently published single-institution study investigated the safety and outcomes of IVC filters in 386 patients, 179 with retrievable filters and 207 with permanent filters. These researchers also found that 20% of the patients with retrievable filters and 24% with permanent filters had recurrent VTEs. The median survival in this study was also consistent with previous literature, with 8.9 months in patients with the retrievable filter group and 3.2 months in the permanent filter group [44]. In addition to the lack of benefits, there are indicators of possible harm. In a large retrospective cohort study that used a population-based sample from two databases, including 126,000 patients, 36.1% were cancer patients. It was shown that in hospitalized patients with VTE and a contraindication to anticoagulation, IVC filter placement was associated with a significantly increased hazard of 30-day mortality (HR = 1.18; 95% CI, 1.13–1.22; *p* < 0.001) [45]. A study utilizing the database of a large urban medical center in Bronx, New York, examined 5263 patients with VTEs. Patients were divided into a group that received anticoagulation only and another group that received an IVC filter in addition to anticoagulation. Both groups showed no significant difference in the percentage of patients with cancer (26.7% versus 30.7%). The study found that patients with IVC filters had increased mortality (HR 1.4, 95% CI: 1.14–1.71, *p* < 0.002). However, this mortality difference disappeared when age, cancer, and albumin levels were adjusted in a multivariate Cox regression model (HR 0.98, CI: 0.79–1.2, *p* = 0.85) [46].

The cost of IVC filters is another issue that should be considered. A study found that a single retrievable filter may cost USD 1576 for the filter itself, USD 10,000 to place, and USD 8824 to retrieve, totaling USD 21,383. The cost of a permanent filter device was USD 4695 and USD 13,289, totaling USD 17,984. The authors’ institution found that over the approximately five-year study period, the total cost of retrievable filters, including their placement and retrieval, which only occurred in 40% of patients, was USD 2,883,389, while the total cost of permanent filters and their placement was USD 3,722,688 for a combined total of over USD 6.6 million for 386 patients in the study [44]. This adds to the substantial financial burden cancer patients face in their final years or months, especially when considering the lack of clear benefits [47].

Our study has certain limitations that must be addressed. First, it was a retrospective study, which could have introduced a bias. However, designing and performing prospective studies and clinical trials addressing this question might be difficult to execute, given the potential harm associated with no IVC filter insertion. Second, the findings of our study may have been affected by the wide range of cancer types and stages present in our sample. Lastly, this was a single-center study, and its findings may not be generalizable. However, our study adds to the growing concerns related to the overutilization of IVC filters in cancer patients with advanced-stage disease, poor prognosis and anticipated short survival. The concept of supportive and palliative care should be emphasized, fully addressed, and discussed with the patients themselves and their family members when appropriate.

In conclusion, the findings of our study suggest that the benefits of IVC filter placement in patients with advanced-stage disease are limited. The complications associated with IVC filters, their lack of benefits, and the financial burden they impose on patients and the healthcare system must be addressed. Therefore, there is a need for prospective studies and large randomized controlled trials tailored to cancer patients. Finally, clinicians should consider the patient’s tumor type and stage, life expectancy, and the presence of proper indications before IVC filter insertion.

## Figures and Tables

**Table 1:** Patient Characteristics.

Clinical Characteristics	Number of Patients	Percentage
Median age (IQR), years	56 (47–65)	
Gender		
Male	86	48.9
Female	90	51.1
Primary Cancer		
Brain	28	15.9
Breast	16	9.1
Colorectal	14	7.9
Lung	13	7.4
Bladder	11	6.3
Leukemia	11	6.3
Lymphoma	10	5.7
Pancreatic	10	5.7
Sarcoma	8	4.5
Prostate	5	2.8
Ovarian	5	2.8
Stage of cancer at the time of IVC insertion		
Early stage	25	14.2
Metastatic	99	56.3
Unstageable (hematological)	24	13.6
Unstageable (brain)	28	15.9
Type of anticancer therapy during the 6 weeks before IVC filter		
Chemotherapy/Immunotherapy	70	39.8
Major surgery	14	7.9
Radiotherapy	10	5.7
Palliative care	41	23.3
Other	36	20.5
Site of VTE		
PE	44	25
Lower extremity DVT	81	46
DVT and PE	41	23.3
Indication for filter insertion		
Contraindication for anti-coagulation	99	56.3
Recurrent VTE while on anticoagulation	56	31.8

IQR, interquartile range; IVC, inferior vena cava; DVT, deep vein thrombosis; PE, pulmonary embolism.

## Data Availability

The data presented in this study are available on request from the corresponding author.
